# Partners’ expectations and experiences of the project ‘Midwife All the Way’: A qualitative study

**DOI:** 10.18332/ejm/136424

**Published:** 2021-06-16

**Authors:** Birgitta Larsson, Li Thies-Lagergren

**Affiliations:** 1 Department of Health Promoting Science, Sophiahemmet University, Stockholm, Sweden; 2 Midwifery Research - Reproductive, Perinatal and Sexual Health, Department of Health Sciences, Faculty of Medicine, Lund University, Lund, Sweden; 3 Department of Obstetrics and Gynaecology, Helsingborg Hospital, Malmö, Sweden

**Keywords:** caseload, continuity, interview, midwifery, partners, rural area

## Abstract

**INTRODUCTION:**

Continuity models of midwifery care are significant factors in facilitating a positive childbirth experience for birthing women. A knowledge gap exists regarding partners’ experiences of continuity of midwifery care during pregnancy, birth, and after birth, although it is essential to understand the experiences of both parents in relation to continuity of care. Thus, the aim of this study was to highlight partners’ expectations and experiences of having participated in a continuity of midwifery care project.

**METHODS:**

A qualitative interview study using thematic analysis was carried out. Thirty-six partners in a rural area in northern Sweden were recruited after the closure of the local labor ward. Interviews were conducted in October 2019 and in May 2020.

**RESULTS:**

An overarching theme: ‘A partner-midwife relationship facilitated a sense of security’; and two themes ‘The concept of availability’ and ‘The midwife’s competence and professionalism’ reflect partners’ expectations and experiences after participating in a continuity of midwifery care project.

**CONCLUSIONS:**

Professionalism was most highly valued, but establishing a relationship with a known midwife facilitated a sense of security. When birthing women feel safe with the known midwife, the partners also feel safe. Having to travel a long-distance to a labor ward caused concern for the partners. This highlights the importance of an organization that supports families to gain access to continuity models of midwifery care and to have a possibility to give birth closer to their residence. The results of this qualitative study further strengthen the growing evidence of the positive effects of continuity models of midwifery care.

## INTRODUCTION

Continuity models of midwifery care are indisputably the most significant factors in facilitating a positive childbirth experience^1^ and result in beneficial obstetric outcomes compared to standard care^2,3^. Standard care is medical or shared care^4^. Continuity models of midwifery care are defined by the World Health Organization (WHO)^5^ as models in which a known and trusted midwife (caseload midwifery), or small group of known midwives (team midwifery) support a woman throughout the antenatal, intrapartum and postnatal period. The WHO recommends that continuity models be accessible for all women and newborns globally and in a recently published scoping review the authors claimed continuity of midwifery care models as the gold standard of maternity care^6^.

In Sweden, midwives are the primary caregivers during normal pregnancy, birth and the first week after birth, here named postpartum period. If complications occur, midwives work in collaboration with obstetricians. Commonly, continuity with the same midwife during pregnancy is high but it is rare that the antenatal midwife assists the woman during birth^7^.

Most often the birthing woman is either married or lives together with a partner; studies show that the partner plays an interactive role and has a desire to be involved in the care given during pregnancy and birth^8,9^. A narrative review describes how some fathers take an active role in providing emotional and practical support whereas others felt themselves to be observers^10^. A majority of partners experience birth in a positive manner and feel included^11,12^. Yet, according to a meta synthesis by Steen et al.^13^, partners often felt excluded resulting in uncertain and fearful feelings. The postpartum period has also been described as a stressful transition period for new fathers^14^. In an interview study regarding continuity of care by midwifery students, it was shown that fathers experienced relational continuity which enhanced empowerment and trust, however, this was mostly important during birth^15^. Overall, there is a knowledge gap regarding partners’ experiences of a known midwife or continuity of care during pregnancy, birth and the postpartum period. There is a rich body of evidence regarding women’s very positive experiences of continuity of care and no potential medical or mental adverse outcomes have been linked to this midwifery model of care^3^. It is of importance to include all parents-to-be in relation to continuity of care(r). Thus, the aim of this study was to highlight partners’ expectations and experiences of having participated in a continuity of midwifery care project.

## METHOD

### Design

This qualitative interview study included partners who participated in a project where couples were offered continuity of midwifery care with a known midwife during pregnancy, labor and the postpartum period.

### Study setting

In 2017, a collaborative-project called Midwife All the Way (MAW) was initiated in a rural area in Northern Sweden. In this geographical area, three labor wards operated, two small units and one larger. After a closedown of the smallest labor ward in February 2017, following a political decision, pregnant women and their partners had, as a consequence to travel 100–120 km to the other hospitals in the area. In 2018, the annual birth rates at the two remaining labor wards were 1866 and 736, respectively.

The closure of the smallest labor ward enabled the start of the MAW project as the midwives who were previously employed in the hospital were potentially available for recruitment to the project; four midwives were subsequently recruited to the project. The midwives provided antenatal care to the study participants and were on-call for births every day, seven days a week between 7 a.m. and 11 p.m. during a 22-month long period, which stretched from 1 August 2017 to 30 June 2019. Sparsity of midwives in the project was the main reason for not being able to have an on-call service 24 hours 7 days per week. The couples were able to reach the midwife on-call via mobile phone with excellent coverage, which was necessary in this rural area. If the couples had been admitted to the labor ward during off hours, the project midwife was to be contacted by the staff at the labor ward at 7 a.m. when the on-call service started. Due to the long distance to the labor wards, the midwives had access to a four-wheel-drive car with the required equipment for emergency births. A more detailed description of the project is presented in Hildingsson et al.^16^.

### Participants and procedure

Partners to women who had participated in the MAW project were included in the present interview study. Initially, recruitment to the MAW project was performed when pregnant women called the antenatal clinic to book an appointment. Information and an offer to participate were given to all Swedish-speaking women and couples. If the woman or the couple consented for participation, they were assigned a primary midwife who performed all antenatal visits. During parental education, or during visits at the antenatal clinic, the couples became familiar with the other project midwives, before the upcoming birth. Following consent for participation, both the woman and her partner answered two questionnaires individually: the first during pregnancy and the second two months after birth. In the questionnaire two months after birth, the couples responded to a question regarding participation in an interview study: ‘May we contact you for a follow-up interview?’. Of the 181 partners who responded to the second questionnaire, 74 (41 %) consented to participate in the interview study and provided their mobile phone number. The partners who had confirmed their willingness to participate were thereafter randomly contacted.

### Data collection

The interviews were conducted in October 2019 and in May 2020 by the last author and two midwifery students. The partners were first contacted by a text message to remind them of their previous consent to be contacted. Brief information was also given and a proposal of date and time for a telephone interview.

The partners all then agreed to be interviewed individually by telephone. Before the first telephone conversation, a pilot interview was conducted with two persons known to the interviewers, in order to familiarize themselves with the question material. An interview guide consisting of semistructured questions, designed to explore the participants’ expectations and experiences of the project, was used. In addition to these questions, four background questions were posed: year of birth, first or subsequent child, educational background and country of birth. The data collection was considered complete after 36 interviews, when no new information emerged. The interviews lasted 27 minutes on average (range: 17–37). They were digitally recorded, transcribed verbatim and anonymized.

### Data analysis

Thematic analysis as described by Braun and Clarke^17^ following six phases of analysis was used. The transcribed interviews were read thoroughly by the two authors to familiarize themselves with data and to identify patterns. The text body was processed successively and the most significant parts of the text were selected and initial codes were generated manually. The coding was discussed and subsequent discrepancies among the codes were further refined. The selected encodings were grouped and formed potential themes and sub-themes. All identified subthemes and themes were discussed between the authors. After being compared, reorganized and refined several times, the final overarching theme, themes and subthemes were defined.

## RESULTS

Of the 36 interviewed partners, 19 had their first child, 14 their second child and three partners their third and fourth child. The partners’ ages ranged from 21 to 54 years. All partners, except one, were male. A majority, 31 partners, had an education at primary or secondary school level and five had a university degree. All partners except one were born in Sweden. Eighteen couples (50%) had been assisted by a known project midwife during birth and the remaining had been assisted by midwives not included in the project during pregnancy, birth and the postpartum. There were various reasons why the couple did not have access to a known midwife during birth: admission to the labor ward out of project hours, project midwife assisted at another birth, the midwife was off work, on holiday, on sick-leave or was not contacted.

The analysis generated the overarching theme: ‘A partner-midwife relationship facilitated a sense of security’, which described how the partners experienced the importance of connecting and creating a relationship with the midwife during pregnancy and during and after the birth of their child. The overarching theme consists of two themes and four sub-themes. The two themes were: ‘The concept of availability’ and ‘The midwife’s competence and professionalism’ (Table 1).

**Table 1:** Overarching theme, themes and subthemes

*A partner-midwife relationship facilitated a sense of security*			
The concept of availability		The midwife’s competence and professionalism	
Access to a known midwife	Safe all the way	Support and communication	A safe woman is a safe partner

### The concept of availability

The theme ‘The concept of availability’ reflects partners’ expectations and experiences of a midwife being within reach near their home whenever it was necessary. They described a feeling of safety knowing that a midwife was reachable, even though they might not have to actually make contact. It was important that it should be the same midwife every time, ‘this sounded sensible’. The theme also reflected the partners’ expectations of the midwife being present when they arrived at the labor ward. The theme was based on two sub-themes labeled: ‘Access to a known midwife’ and ‘Safe all the way’.

### Access to a known midwife

The most prominent expectation of participating in the project was to feel secure. Several partners stated that due to a complication-free pregnancy it had not been necessary to make contact, but the mere awareness of the possibility to access to a known midwife provide extra security. This possibility resulted in reduced stress and nervousness. Some partners expressed that even though the opportunity to call the midwife was restricted to the hours between 7 a.m. and 11 p.m., it had been extra valuable, and the partners expressed gratitude for the opportunity offered. Accessibility to a known midwife close to home was described as very important:

*‘We did not have much contact but it was good that she was close if something should happen’.*

Many of the partners did not have their expectations met regarding access to a known midwife during pregnancy, birth and postpartum. Having had a known midwife was experienced as very positive and it was considered by some as a ‘luxury’ to have created a trusting relationship with the midwife:

*‘So, she got there, and it was a huge luxury because she was in the room all the time. So, it was kind of luxurious.’*

To connect and create a relationship with the midwife was highly valued:

*‘I think it's very important. Creating a relationship with someone who is involved in one's life is vital. When you have a relationship with a person who knows your needs and wishes, you become more relaxed.’* (Alexander, first child)

After the birth it was important to meet the midwife who had been involved in the whole process. The partners expressed positive feelings when the postpartum follow-up was with someone they knew and trusted. It was particularly important if an event had required special processing. Some expressed that having access to a known midwife after the birth was of great importance for starting up family life:

*‘Initially Elin had a problem with breastfeeding and then she [the midwife] came and gave some tips and advice - that was safe - midwife all the way.’*

Despite having access to a known midwife some expectations were not met:

*‘It never became such a relationship, no!’*

Some also had experienced that accessibility was reduced. One partner described how nobody answered the on-call telephone number and they had to call the labor ward instead, making inclusion in the project futile. It happened that participation in the project did not meet expectations in the long run:

*‘This whole thing was huge from the beginning, they were on call and you could call around the clock. But then it was stepped down because there were not enough midwives, and then they only had a phone between 7 and 4, but if there was a problem or something you were wondering about, you could call and check with them [during the given time frame]. A little security, it was a security, though it fizzled out. There was not much at the end.’* (Christian, first child)

It was also expressed that a known midwife was not important if and when making contact: *‘… that our midwife was not available didn't affect the experience.’*

### Safe all the way

Another expectation was that they should not feel alone when birth started as this would decrease stress and was an extra security especially for first-time parents, giving them the opportunity for telephone contact for advice. The partners expressed a hope that the midwife would have time to accompany them all the way from their hometown to the labor ward and throughout the birth. It was described how the midwife drove behind the couple from home to hospital and once arriving at the labor ward, assisted at the birth:

*‘We called the midwife, so she packed her bag right away and drove off.’*

Nervousness was expressed about the new and unknown situation and the telephone contact was perceived as a security and bonus for the prospective parents:

*‘... yes, but it's because you want that, that security again. That you had a midwife who had been with you since day 1 and all the way. Obviously, for our sake, we wanted that she [the midwife] would be there [on the road] as well…’* (David, first child)

The mere thought of the journey from home to the hospital and being alone on the road created anxiety and fear in some of the partners:

*‘There were no worries about the [birth] process, there was more worry about giving birth on the road.’*

One partner described how he felt when things did not turn out as expected:

*‘It sounded very positive to have the same midwife all the way, but that did not happen. I expected to meet the same midwife every time and feel safe with her and even when we should drive down to the hospital, she should be behind in her car or in front. So, we had no back-up, in front or behind on the journey. Yes, that was the thing. That you would get to meet up before and go together. Not the same car, but so they were behind. So, you know that there is someone who can help. To be honest, I overtook on an unbroken white line, I drove on the wrong side of the traffic island. My partner roared in the car. We said all the time that the police can't be angry at us. I crossed a red light when we came to the town …’* (Erik, second child)

Other partners expressed that they felt all right without a midwife being physically accessible on their journey to the hospital:

*‘Well it was calm, I actually drove very calmly and my partner was self-controlled. We had 120-130 km ahead of us, but I packed what was needed if there was to be a birth in the car but it went well. I thought it could be (car birth) - she has had many before and those births had often gone fast.’* (Filip, first child)

It was both important and unimportant with a known midwife on the journey to the labor ward.

### The midwife’s competence and professionalism

This theme mirrors the partners’ experiences of midwives’ competence and professionalism. All partners showed great confidence in the midwives’ ability to support their spouse whether it was a midwife they knew beforehand or a complete stranger:

*‘It does not matter if the midwives are known as long as they are competent.’*

Some partners expressed that confidence in the profession diminished the importance of a known midwife. The theme was based on the sub-themes: *‘Support and communication’ and ‘A safe woman is a safe partner’.*

### Support and communication

By joining the project, the partners expected individualized and customized midwifery support. The partners expressed that they wanted to have a known midwife, as she would know what was important for the couple. In the case of fear of childbirth, for example, the project was seen as an opportunity for more customized support. Also, a hope was expressed that participation in the project would alleviate various concerns:

*‘Our midwife was a specialist in this so it was nice to have her …. Yes, it felt better because she knew us and we her. We could talk about everything.’*

By having access to a known midwife, they hoped for a closer and friendly contact. When a relationship was preestablished, it was easier to communicate with the midwife and the partners expressed that the midwife included them to a great extent when communicating during the birth process:

*‘The midwife was a great support for us and she talked a lot with me during the birth.’*

For some partners it was not important to have a known midwife because they felt supported anyway. Competent staff allowed the partner to focus on the birth and the midwife’s personal approach was more important than if the midwife was known. Some expressed that the confidence in the profession diminished the importance of a known midwife:

*‘As long as they know their stuff, it really does not matter who helps.’*

### A safe woman is a safe partner

The partners expressed being very focused on the woman’s feelings of safety during pregnancy, birth and postpartum. They expressed, that for the sake of the woman, things would feel safer if they had a known midwife:

*‘If she feels safe, I feel safe. So, if she trusts the situation, she trusts me on it too.’*

Partners also saw having access to a known midwife as a ‘win-win’ situation. One partner said:

*‘A happy woman gives a happy man. If she's happy with the situation, I'm happy with it.’*

It was also expressed when the mother felt safe it could relieve their own stress:

*‘If you look at it purely egocentrically, it might have been more stressful for my wife [not knowing the midwife] and then it would have been more stressful for me who is already in a stressful situation.’* (Gustaf, second child)

## DISCUSSION

As far as the authors have been able to ascertain, this qualitative interview study is the first study describing partners’ expectations and experiences of participation in a continuity of midwifery care project in a Swedish rural context. Those included in the study described that availability of a midwife, her competence and professionalism were more important than the actual presence of a known midwife at birth. Having said this, the partners expressed that knowing the midwife facilitated a relationship, which in turn facilitated a sense of security. The partners described a feeling of being included in the process during pregnancy, childbirth and the postpartum period, and this was highly valued. It can be argued that a known midwife can facilitate this feeling of inclusion, thus counteracting feelings of disconnection with their partner’s pregnancy and labor, which has previously been described by Longworth and Kingdon^18^.

The partners highlighted the importance of the midwives’ availability by phone at the onset of labor or if questions arose. Preferably it should be a known midwife and this is in line with results in a qualitative study by Jepsen et al.^19^ where couples described easy access to their known midwife as helpful and calming. In addition, they expressed experiencing a close and personal contact compared to talking to a random midwife at the labor ward. A growing body of evidence shows that women prefer continuity models of midwifery care which enable accessibility to a known midwife^1,3,20^. There is no reason to believe this fact should be any different for partners than for birthing women.

The mere possibility to have access to a known midwife provided extra security. In addition, the partners expressed how important it was to meet their known midwife after birth and how happy they felt to be able to tie it all together. In contrast to these positive expressions, some partners were disappointed at not experiencing the continuity with their midwife that they expected from their participation in the ‘Midwife All the Way’ project. It is likely that lack of information regarding the model and what to expect caused this result. It is not possible to reach 100% continuity of care by a known midwife. In earlier studies, 75–80% of women or couples in continuity models with on-call service (24/7) are assisted by a known midwife during birth^19,21^. It is of great importance to make clear to participants in projects of this kind what they might expect from a continuity model.

Interestingly, partners expressed that the expectations of participating in the project were twofold: firstly, knowing who to contact when needed and secondly the anticipated feeling of safety when a midwife accompanied the couple on the long road journey from home to the labor ward, 120 km away. Most couples, however, did not have their midwife with them along the road and several partners expressed that they had hoped she would be close at hand if the woman should give birth during the journey. Many felt greatly worried about the long drive, which could be in heavy weather conditions with bad phone coverage. Some understood and accepted the fact that they had to drive alone, while others presumed that accompaniment had been the purpose of the project. Some partners interpreted the project ‘Midwife All the Way’ literally and this misunderstanding may also be due to a lack of clear information.

It can be argued that it is of great importance to listen to the voices of birthing women and their partners about their request of access to continuity of midwifery care in their local surroundings. In an Australian study, the importance to women who live in very remote areas of having the opportunity to give birth close to their residence with midwife-led care was acknowledged by clinicians and health policy-makers^22^. In Sweden, the midwife is the primary caregiver during pregnancy, labor and the postpartum, albeit within a state funded, centralized, medical-led model of care^23^. In an explorative and descriptive qualitative study, it was found that obstetric discourse promoting the benefits of birthing in a centralized unit ignores the risks of travelling long distances during labor^24^. It is evident that women assessed as low risk who are cared for by a midwife within midwifery-led units are safe^24^ and thus such organizations should be offered in rural areas.

Our results revealed that a known midwife was important to the partners, particularly as contact and communication during pregnancy and the postnatal period can be facilitated. However, during birth the midwife’s professionalism and skill took precedence. In contrast, a Norwegian qualitative study of fathers’ expectations of continuity of midwifery students, pointed out that a continuous relationship affected them positively during birth, but was experienced as being of less importance during pregnancy and the postpartum period^15^.

When a relationship is created during pregnancy it may pave the way for partners to experience teamwork with the midwife and the woman during labor and postpartum. Partners in the present study valued the fact that a continuity of midwifery care model could offer them support and communication thus assail feelings of exclusion. This is in line with Jepsen et al.^19^ who described fathers’ experiences of a continuity of midwifery care organization which enhanced feelings of trust and confidence.

The partners in the present study described how their focus on the women’s wellbeing took precedence over their own needs and wishes, a finding which has previously been described by Johansson et al.^25^. It was also shown here that regardless of whether the partner created a relationship with the midwife, the woman’s feelings of safety were paramount, a finding also captured by Jepsen et al.^19^ who described how partners in a midwifery care study felt that the woman was able to transmit positive feelings to them and that they trusted her discernment.

### Strengths and limitations

Several aspects have been taken into consideration in order to enhance trustworthiness of the results in this study^26^.

The relatively many respondents, the equal distribution between couples who had a known midwife during birth versus those who did not, strengthen the credibility of the study. In addition, frequent debriefing sessions between the authors during the research process further strengthen the credibility. However, when responding to the question of a follow-up interview two months after birth, partners who have had access to a known midwife might have been more supportive to the model thus responded to a greater extent. This could have influenced the result.

The rural context in which this study took place may render the findings inapplicable to a wider population. Further research should include partners in an urban setting as a vast majority of babies are born in bigger towns or large cities. However, the description of the study context, data collection and analysis of data may enable other researchers to evaluate the relevance of the study and the transferability of the methods.

It has been shown that level of education is an essential and powerful explanatory factor in the choice of health care^27^. Less than 14 % of the partners in this study had a university degree, which is a weakness since perspectives from partners of higher socioeconomic background are not included. It would be of interest to perform a similar study in a cohort of fathers or partners within higher socioeconomic backgrounds as previous studies have shown that women, despite level of education, benefit from continuity of midwifery models of care^16,28^.

The interviews were all conducted via telephone due to the rural setting which would have entailed long travelling distances for the researchers had the interviews been conducted in person. This could have affected how the respondents answered when compared to face-to-face interviews^29^. However, interviews by telephone may allow respondents to talk more freely^29^ and it is time-saving, which allows for more interviews.

The methodological description of the study enhances dependability; confirmability was enhanced by using the Braun and Clark^17^ checklist of criteria for good thematic analysis. In addition, both authors are midwives with clinical experience of maternity care. Throughout the interviews and analyses, the authors paid careful attention to their pre-understanding that would inevitably influence the interpretation of the partner’s stories.

## CONCLUSIONS

Partners expected to be accompanied and supported by a known midwife when participating in the project ‘Midwife All the Way’. Less than half of the participants experienced having a midwife all the way. It was shown that professionalism was the most valued and important quality in a midwife. However, establishment of a relationship with a known midwife facilitated a sense of security. When birthing women feel safe with the known midwife, the partners also feel safe. The long travelling distance to labor wards was of great concern for the partners. This highlights the importance of an organization which supports access to continuity models of midwifery care and creates the possibility for women to give birth closer to their residence. The results of this qualitative study further strengthen the growing evidence of the positive effects of continuity models of midwifery care.

## Data Availability

The data supporting this research cannot be made available for privacy reasons.
